# Common Denominators in the Immunobiology of IgG4 Autoimmune Diseases: What Do Glomerulonephritis, Pemphigus Vulgaris, Myasthenia Gravis, Thrombotic Thrombocytopenic Purpura and Autoimmune Encephalitis Have in Common?

**DOI:** 10.3389/fimmu.2020.605214

**Published:** 2021-01-29

**Authors:** Inga Koneczny, Vuslat Yilmaz, Konstantinos Lazaridis, John Tzartos, Tobias L. Lenz, Socrates Tzartos, Erdem Tüzün, Frank Leypoldt

**Affiliations:** ^1^ Division of Neuropathology and Neurochemistry, Department of Neurology, Medical University of Vienna, Vienna, Austria; ^2^ Department of Neuroscience, Aziz Sancar Institute of Experimental Medicine, Istanbul University, Istanbul, Turkey; ^3^ Department of Immunology, Laboratory of Immunology, Hellenic Pasteur Institute, Athens, Greece; ^4^ Tzartos NeuroDiagnostics, Athens, Greece; ^5^ 1st Department of Neurology, Eginition Hospital, National and Kapodistrian University of Athens, Athens, Greece; ^6^ Research Group for Evolutionary Immunogenomics, Max Planck Institute for Evolutionary Biology, Plön, Germany; ^7^ Department of Neurobiology, Hellenic Pasteur Institute, Athens, Greece; ^8^ Neuroimmunology, Institute of Clinical Chemistry and Department of Neurology, Medical Faculty, Christian-Albrechts-University Kiel, Kiel, Germany

**Keywords:** IgG4 autoimmune disease, MHC, autoimmunity, HLA-DRB1, HLA-DQB1, etiology, HLA class II

## Abstract

IgG4 autoimmune diseases (IgG4-AID) are an emerging group of autoimmune diseases that are caused by pathogenic autoantibodies of the IgG4 subclass. It has only recently been appreciated, that members of this group share relevant immunobiological and therapeutic aspects even though different antigens, tissues and organs are affected: glomerulonephritis (kidney), pemphigus vulgaris (skin), thrombotic thrombocytopenic purpura (hematologic system) muscle-specific kinase (MuSK) in myasthenia gravis (peripheral nervous system) and autoimmune encephalitis (central nervous system) to give some examples. In all these diseases, patients’ IgG4 subclass autoantibodies block protein-protein interactions instead of causing complement mediated tissue injury, patients respond favorably to rituximab and share a genetic predisposition: at least five HLA class II genes have been reported in individual studies to be associated with several different IgG4-AID. This suggests a role for the HLA class II region and specifically the DRβ1 chain for aberrant priming of autoreactive T-cells toward a chronic immune response skewed toward the production of IgG4 subclass autoantibodies. The aim of this review is to provide an update on findings arguing for a common pathogenic mechanism in IgG4-AID in general and to provide hypotheses about the role of distinct HLA haplotypes, T-cells and cytokines in IgG4-AID.

## Introduction to IgG4-AID

**Figure 1:**
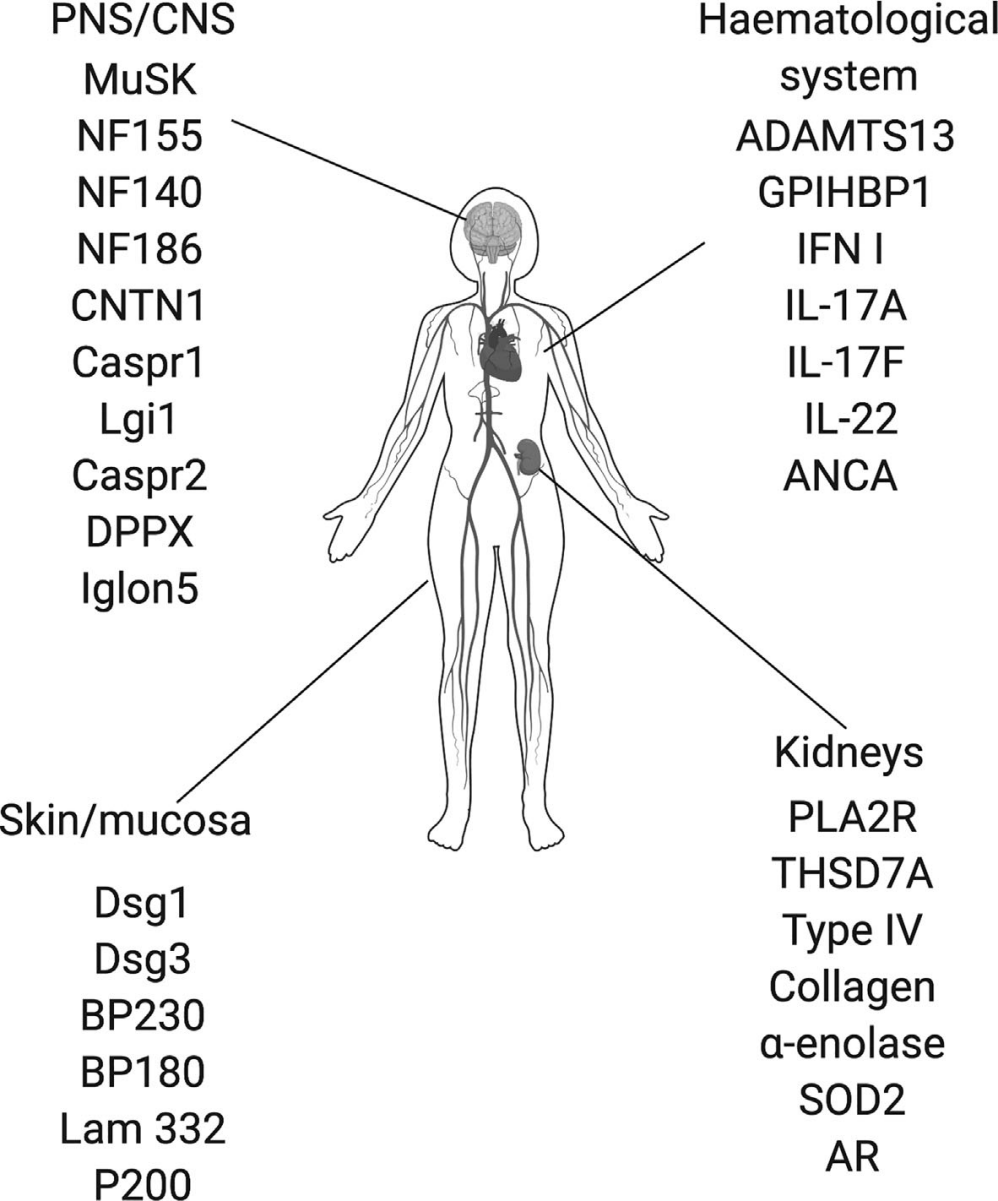
Proposed IgG4 autoantibody targets. ADAMTS13, disintegrin and metalloproteinase with thrombospondin motifs 13; ANCA, antineutrophil cytoplasmic antibodies; AR, Aldose reductase; BP180; BP230, bullous pemphigoid antigen 180/230; CNTN1, contactin 1; Caspr1/2, contactin associated protein1/2; Dsg1/3, desmoglein 1/3; DPPX, dipeptidyl-peptidase-like protein 6; GPIHBP1, glycosylphosphatidylinositol-anchored high density lipoprotein–binding protein 1; IFN I, type I interferon, IgLON5, IgLON family member 5; IL-17A/17F/22, interleukin-17A/17F/22; Lam332, Laminin 332; LGI1, leucine-rich, glioma inactivated 1; MuSK, muscle-specific kinase; NF140/155/186; Neurofascin 140/155/186; PLA2R, phospholipase A2 receptor; SOD2, manganese superoxide dismutase; THSD7A, thrombospondin type-1 domain- containing 7A.

IgG4 autoimmune diseases (IgG4-AID) are a group of autoimmune diseases that are mediated by antigen-specific autoantibodies of the IgG4 subclass (1, 2). The best known IgG4 autoimmune diseases include pemphigus (3), MuSK myasthenia gravis (4), thrombotic-thrombocytopenic purpura (5), Goodpasture syndrome (6) and membranous nephropathy (7, 8). IgG4 is the least common subclass of IgG, it is immunologically inert as it does not activate complement or engage activating Fcγ receptors, which is also why therapeutic monoclonal antibodies are frequently modeled on the IgG4 subclass (9, 10). Since IgG4 is also produced in response to strong or chronic stimulation with antigen to down-regulate an overshooting immune response, it is often considered a protective antibody subclass, competing with other antibody classes and subclasses, e.g. IgG1 or IgE, for binding of antigen [reviewed here (9, 11, 12):]. Therefore, IgG4 is not considered as a pathogenic player in autoimmunity, and is an unlikely antibody to cause harm. Yet in recent years, more and more diseases were found to be primarily associated with IgG4 subclass autoantibodies targeting different autoantigens (2, 13). IgG4 autoimmune diseases were first appreciated as a group in 2015 (13). The number of suspected IgG4-AID is constantly growing and to date, 29 candidate antigens are known [**Figure 1**, (14)]. Most antigens are located in the central and peripheral nervous system, but there are also antigens in the skin and mucosa, kidneys and in the hematological system (**Figure 1**). The group of IgG4-AID is defined by several characteristics beyond mere antibody isotype predominance: 1) the IgG4 autoantibodies target transmembrane/extracellular or soluble antigens, 2) are directly pathogenic, meaning that they exert their effect without complement- or cell-dependent cytotoxicity by blocking of binding sites for direct interaction partners, ligands or substrates, 3) all IgG4-AID have a low prevalence affecting less than 5 per 10,000, and 4) mostly respond favorable to treatment with the B-cell depleting agent rituximab, an observation that has been puzzling because long-lived antibody producing plasma cells do not carry CD20 and are therefore not depleted. Another more recent observation points into the same direction of IgG4-AID sharing common immunobiological mechanisms: Most of them are associated with similar variants of the HLA class II region (**Table 1**). In this review, we summarize the information available for the group of IgG4-AID and hypothesize on common immunopathogenesis.

**Table 1:** Reported HLA-associations of IgG4 autoimmune diseases.

Disease/antigen	Affected organ	Reported HLA association	DQB1*05	DRB1*04	DRB1*11	DRB1*14	DRB1*15	Protective alleles/No association	References
Class I IgG4 autoimmune diseases									
MuSK-MG/MuSK	PNS	DQB1*05, DRB1*14, DRB1*16, DQB*03, DQB1*0502, DQB1*0301/0313, DQA1*0101 (Iran)	✓			✓		DQA1* alleles (except Iran) HLA-DRB1∗03 (Turkey) DRB1*13 (Serbia)	(15–23)
Pemphigus foliaceus, fogo selvagem (and p. vulgaris of mucocutaneous type)/Desmoglein 1	Skin/mucosa	PF: HLA-DRB1*04, HLA-DRB1*14, DRB1*1404 DQB1*0503, HLA-A10 FS: DRB1*01 DRB1*0404, *1402, *1406 or *0102	✓	✓		✓		N/A	(24–30)
Pemphigus vulgaris/Desmoglein 3	Skin/mucosa	HLA-DRB1* 0402, HLA-DRB1*1401/*1402, HLA-DQB1*05:03, HLA-DQB1*03 HLA-DRB1*03 DQB1*03:02, DQB1*0503	✓	✓		✓		DQB1*05:01, DQB1*02, DQB1*06:01, and DQB1*03:03	(27–29, 31, 32)
Thrombic thrombocytopenic purpura/ADAMTS13	Blood/hematological system	HLA-DRB1*11, HLA-DRB1*04, HLA-DRB1*14, HLA-DRB4, HLA-DQB1*03, and HLA-DQB1*05/*06/*02, HLA-A*03 DRB1∗15, HLA-DQB1*02:02	✓	✓	✓	✓	✓	HLA-DRB1*03DRB1*07-DQB1*02 and DRB1 *13-DQB1 *06 HLA-DRB1*04 (John study)	(33–37)
CIDP/Neurofascin 155	PNS	HLA-DRB15*01 and *02					✓	N/A	(38)
CIDP/CNTN1 (Contactin 1)/	CNS	N/A	N/A	N/A	N/A	N/A	N/A	N/A	N/A
Class II IgG4 autoimmune diseases									
Encephalitis Morvan’s syndrome/LGI1	CNS	HLA-DRB1*07:01 and linked alleles (HLA-DRB1*07:01- DQB1*02:02) HLA-DR7 and HLA-DRB4 HLA-B*44:03, C*07:06		✓				N/A	(39–42)
Membranous nephropathy/PLA2R	Kidneys	DRB1*15:01, DRB3*0202,					✓	N/A	(7, 43, 44)
CNS and PNS disorders/CASPR2	CNS/PNS	HLA-DRB1*11:01 (Linkage: DRB1*11:01-DQA1*05:01-DQB1*03:01)			✓			N/A	(39)
Membranous nephropathy/THSD7A	Kidneys	N/A	N/A	N/A	N/A	N/A	N/A	N/A	N/A
GPIHBP1 autoantibody syndrome/GPIHBP1	Blood/hematological system	N/A	N/A	N/A	N/A	N/A	N/A	N/A	N/A
CIDP/CASPR1	PNS	N/A	N/A	N/A	N/A	N/A	N/A	N/A	N/A
Mucous membrane pemphigoid/Laminin 332	Skin/mucosa	Unclear/N/A	N/A	N/A	N/A	N/A	N/A	N/A	N/A
Class III IgG4 autoimmune diseases									
Goodpasture syndrome/Type IV collagen	Kidneys	HLA-DRB1*15:01 (DRB1*0404) DRB1∗15:02		✓			✓	N/A	(6, 45–48)
IgLON5 Parasomnia/IgLON5	CNS	HLA-DRB1*10:01, HLA-DQB1*05:01	✓					N/A	(49)
Bullous pemphigoid/Bulloid pemphigoid antigen 180/230	Skin/mucosa	DQB1*0301, DRB1*04, DRB1*11, DQB1*0501 and DRB1*1001, DQA1*05,	✓	✓	✓			HLA-DQA1*01:02/03, HLA-DQB1*02:02, and HLA-DRB1*07:01	(50–53)
Granulomatosis with Polyangiitis/ANCA	Blood/hematological system	HLA-DPB1*0401, HLA-DR1, HLA-DR4, HLA-DRB1*0901-HLA DQB1*0303, HLA-DR9, HLA-DR13, HLA-DRB1*1202, HLA-DRB1*15, HLA-DRB1*0405		✓			✓		(54–56)
Autoimmune polyendocrine syndrome type 1 (APECED)/IFN I, IL-17A, IL-17F and IL-22	Blood/hematological system	DRB1*03:01, DRB1*04:01, DQA1*03:01, DQA1*05:01, DQB1*02:01, DQB1*03:02 Subtypes: Addison’s disease: HLA-DRB1*03, Alopecia: HLA-DRB1*04- DQB1*0302					✓	Subtype with type 1 diabetes: HLA-DRB1*15-DQB1*0602	(54–56)
CIDP/Neurofascin 140/186	PNS	N/A	N/A	N/A	N/A	N/A	N/A	N/A	N/A
Anti-laminin γ1/Anti-P200 pemphigoid/P200 (laminin γ1)	Skin/mucosa	N/A	N/A	N/A	N/A	N/A	N/A	N/A	N/A
DPPX encephalitis/DPPX	CNS	N/A	N/A	N/A	N/A	N/A	N/A	N/A	N/A
Membranous nephropathy/αenolase, SOD2, AR/	Kidneys	N/A	N/A	N/A	N/A	NA/	N/A	N/A	N/A

Notably the study populations are from various different geographic locations and contain participants of diverse ethnicities, which may influence HLA associations. CIDP, chronic inflammatory demyelinating polyneuropathy; N/A, data not available; CNS, central nervous system; PNS, peripheral nervous system.

## Known IgG4 Autoantibody-Associated Disease: A Colorful Bouquet

There are many distinct forms of IgG4-AID with a wide variety of symptoms, caused by IgG4 autoantibodies that may target one of four main organs: 1) the skin and mucosa, 2) the peripheral and central nervous system, 3) the kidneys and 4) the hematological system (**Figure 1**). The pathogenic mechanism of these antibodies is based on an IgG4-mediated blocking of protein-protein and/or cell-cell interaction. The detailed description of all IgG4 -AID and their disease mechanisms would by far exceed the scope of this review and was therefore published separately (14), but we would like to briefly summarize the key mechanisms of a few example diseases.

Probably the most commonly known IgG4-AID targets skin and mucous membranes: pemphigus vulgaris. This disease is hallmarked by acantholysis and blister formation caused by the loss of keratinocyte adherence. The targets of IgG4 autoantibodies are the desmogleins (Dsg1 or 3) located at the desmosomes. Desmogleins interact closely with each other to maintain the adherence of the cells, and thereby the tissue architecture of skin and mucosa. The binding of IgG4 to desmoglein 1 or 3 leads to a loss of interaction between desmogleins, which causes the disruption of the cell adhesion and in consequence to the formation of blisters (57–59).

Some IgG4-AID cause central and/or peripheral nervous system dysfunction, depending on the target antigen localization. Antibodies targeting the CNS synaptic scaffolding protein LGI1 disrupt synaptic signaling by blocking the trans-synaptic clustering of receptors and channels mediated by this antigen. This leads to amnesia, epileptic seizures and psychiatric symptoms (60–62).

In the peripheral nervous system, IgG4 autoantibodies may, for example, recognize the protein neurofascin 155 (NF155). NF155 is important for attaching the myelin sheath lamellae to the axon at the paranodes adjacent to the nodes of Ranvier. This leads to disruption of action potentials (conduction slowing and block) and consecutively to chronic neuropathies [chronic inflammatory demyelinating polyneuropathy; CIDP (63–67)]. In IgG4-AID, which target synaptic antigens localized in the peripheral and central nervous system, e.g. CASPR2, combinations of central nervous system dysfunction (limbic encephalitis) and peripheral nervous system dysfunction (neuromyotonia, pain) may occur (68–70).

The signal for muscle contraction is transmitted by the release of the neurotransmitter acetylcholine from the motoneuron to the muscle, where it is bound by acetylcholine receptors (AChR). Muscle-specific kinase (MuSK) is a key regulator of the neuromuscular junction that is part of a signal transduction pathway required for dense clustering of acetylcholine receptors (AChR) at the neuromuscular junction. IgG4 autoantibodies against MuSK block its interaction with another protein in the signal transduction pathway, leading to reduced densities of AChR (71, 72). As a consequence, there is less efficient neuromuscular transmission. This means that not every signal from the motoneuron to move the muscle leads to a muscle contraction, which translates into a severe, fatigable weakness of skeletal muscles.

Autoantibodies against kidney antigens PLA2R andTHSD7A are found in patients with membranous nephropathy, where the pathogenic mechanisms are still unclear, PLA2R and THSD7A are expressed in cells of the glomerulus (podocytes) inside of renal corpuscles. The glomerulus filters the blood, so the adhesion between the cells is important for the function of the glomerulus, but we know very little about the role of PLA2R and THSD7A and what the autoantibodies exactly do. THSD7A-IgG4 was hypothesized to block protein-protein interactions of THSD7 and cell-cell interactions, therefore affecting the glomerular filtration and causing proteinuria (73–75). Potentially affected binding partners of PLA2R are collagen IV (76), which is important for cell adhesion, or the At2-complex (14, 77), which is important to maintain cell architecture and affect podocyte function. Perhaps a blocking of PLA2R interaction with these binding partners affects cell adhesion and/or cell architecture. Interestingly it is also thought that a specific subtype of complement activation, *via* mannose-binding lectins may occur (78–80).

Thrombotic thrombocytopenic purpura (TTP) is another well-known disease that is mediated by IgG4. Patients with TTP have IgG4 autoantibodies against a protease located in the blood, ADAMTS13. The normal function of ADAMTS13 is to cleave and inactivate the multimeric forms of the von Willebrand factor (vWF), which is a protein that is (in its multimeric form) required for blood clotting, e.g. following injury. Blocking of ADAMTS13 function by IgG4 leads to accumulation of multimeric vWF, which then binds to platelets. This leads to the formation of microthrombi that may block capillaries and therefore cause vascular occlusion. This is especially severe if the thrombi occur in brain, kidneys or lungs (17, 81, 82). A different antigen in the hematological system is GPIHBP1, which is a protein expressed in capillary endothelial cells and which have the main function to transport lipoprotein lipase (LPL) into the blood. LPL breaks down lipids, so when it is not transported into the blood because IgG4 binding to its transporter GPIHBP1, the patients will suffer from hypertriglyceridemia (83, 84).

## Role of HLA Class II Polymorphism in IgG4-AID: Emerging Genetic Predisposition

**Figure 2:**
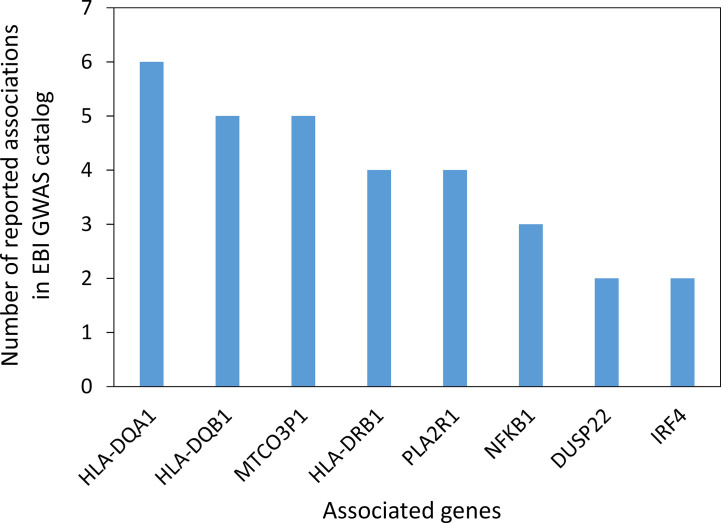
Genes with potential association to IgG4-AID reported in the EBI GWAS catalog (85). Data represents 17 studies investigating four Ig4G-AIDs (Pemphigus vulgaris, Pemphigus foliaceus, Membranous glomerulonephritis, Eosinophilic granulomatosis with polyangiitis; median of 4 studies per disease). The GWAS catalog reports multiple associations per disease and multiple genes mapped to the association signal, so reported genes do not necessarily represent independent associations. The gene MTCO3P1 is a pseudogene located next to HLA-DQB1 in the HLA class II region. Only genes reported more than once are shown. [GWAS catalog accessed on 03.11.2020).

In the search for common immunopathogenesis, we wanted to investigate mutations or variants of genes that may contribute to a genetic predisposition for the development of IgG4-AID. We therefore investigated whether there are candidate genes that have been identified in genome wide association studies (GWAS) of individual IgG4-AID. Data on pemphigus vulgaris, pemphigus foliaceus, membranous glomerulonephritis and eosinophilic granulomatosis with polyangiitis were identified in the NHGRI-EBI catalog of human genome-wide association studies (85). The most frequently reported associations were with variants in the HLA class II region: HLA-DQA1, HLA-DQB1, HLA-DRB1 (**Figure 2**, **Supplementary Table 1**).

Most autoimmune diseases are known to have a genetic association with the HLA class II region, and it is thought that the presence of specific HLA alleles may cause a genetic predisposition to develop specific autoimmune diseases or groups of autoimmune diseases (86–88). Therefore, we also looked at reports of associations with HLA class II genes, as it may be possible that there are one or more HLA alleles that may be specifically associated with one or more IgG4-AIDs. Interestingly, we observed that many studies reported a genetic association of individual IgG4-AID with HLA-class II variation: HLA*DRB1*14, 04,15 or 11, and/or HLA-DQB1*05 (**Table 1**).

For example, the HLA-DRB1*14 supertype was found associated with MuSK MG, pemphigus and thrombotic thrombocytopenic purpura, HLA-DQB1*05 in Iglon5 parasomnia, thrombotic thrombocytopenic purpura and MuSK MG (15, 16, 18, 19, 89), HLA-DRB1*15 is associated with NF155 antibody positive CIDP, pemphigus and PLA2R antibody positive MN [in a Chinese population, up to 99% of patients with PLA2R antibodies carried either DRB1*15:01 or DRB3*02:02, and individuals with these alleles have a 99-fold increased risk of PLA2R-associated MN (43)]. Alleles of the DRB1 and DQB1 genes were associated with most IgG4 autoimmune diseases, suggesting that the HLA class II genes play a role not only in typical autoimmune diseases, but also in the induction of an IgG4 specific autoimmune response. The next question is, whether there is a specific allele associated with a class switch toward IgG4. Looking at IgG4-related diseases in comparison, also the HLA-DRB1 locus was found to be associated in two out of six studies (90), in one of these studies HLA-DRB1*15 was associated with increased IgG4 levels (91). Whether specific alleles are truly associated with an increased predisposition to induce a class switch toward IgG4 and to develop IgG4-AID cannot yet be concluded, but a systematic review and meta-analysis are currently being conducted in our research groups to address this possibility.

## A Potential Explanation for the Gradient: Association of Prevalence and Genetic Background in Europe

**Figure 3:**
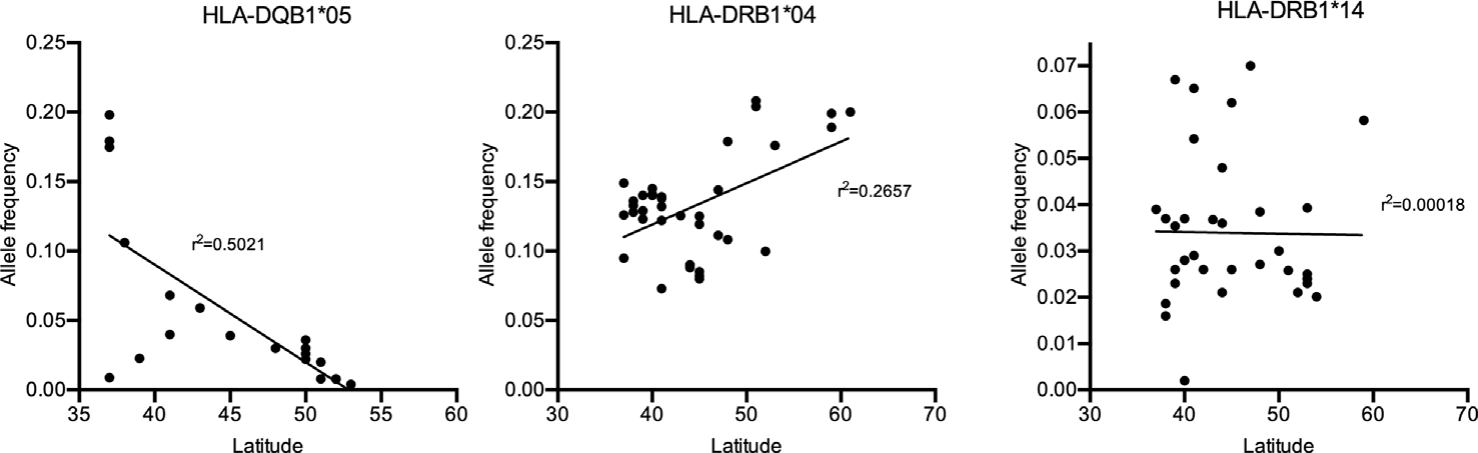
Allele frequency of the HLA-DQB1*05:02 allele in Europe [Source: The Allele Frequency Net Database (AFND)].

If specific HLA class II alleles would contribute to a genetic predisposition for IgG4-AID, it may be possible that geographical differences in HLA class II genotype may correlate with an increased disease prevalence. Interestingly, both in MuSK-MG (92) and in pemphigus vulgaris (93), a north-south gradient can be observed in Europe, with higher prevalence in southern countries such as Italy and fewer cases in northern countries such as Finland or Norway. Potential reasons may be environmental or genetic factors. If there were also geographical differences in HLA class II genotype frequencies, it would underline the relevance of the HLA class II alleles on disease susceptibility. To test this hypothesis, we have plotted the frequency of the HLA-DQB1*05:02 allele as well as of HLA-DRB1*04 and *014 {data obtained from the Allele Frequency Net Database [AFND, (94)], **Table 2**} against latitude in European countries (**Figure 3**). There is a trend toward increased frequency of HLA-DQB1*05:02 in countries with lower latitude, such as Italy, which also show increased frequency of MuSK MG (92) and pemphigus (93), while there is a reduced frequency for HLA-DRB1*04 and no apparent change in HLA-DRB1*14 in countries with low latitudes.

**Table 2:** Frequency of the HLA-DQB1*05:02 allele in European countries sorted by latitude. Source: The Allele Frequency Net Database [AFND (94)].

Country/Region (defined by AFND)	% of population carrying allele	Allele frequency	Cohort size	Geographical location
Ireland Northern pop 2	0.8	0.004	122	54_32_N_5_55_W
Ireland South	1.6	0.008	250	53_20_N_6_15_W
Netherlands UMCU	1.6	0.0078	64	52_5_N_5_10_E
Netherlands	5.5		447	52_0_N_7_0_E
England pop 3	4	0.02	61	51_30_N_0_7_W
England pop 6	1.7	0.008	177	51_30_N_0_7_W
Germany Essen	5.2	0.026	174	51_27_N_7_0_E
Czech Republic pop 3	7.2	0.036	180	50_7_N_14_24_E
Belgium	6.1	0.03	99	50_50_N_4_21_E
Belgium pop 2	4.5	0.022	715	50_50_N_4_21_E
Czech Republic pop 2	5.1		99	50_5_N_14_28_E
Czech Republic Romani	17.6		34	50_5_N_14_28_E
Germany pop 3	3.6		111	50_48_N_8_46_E
Austria	6	0.03	200	48_13_N_16_21_E
France West	3		100	48_0_N_2_0_W
Slovenia pop 2	8.6		140	46_3_N_14_30_E
France Southeast	7.7	0.039	130	46_0_N_5_0_E
Italy Bergamo	11.9	0.059	101	45_42_N_9_40_E
Italy North pop 3	13.4	0.068	97	43_0_N_12_0_E
Italy Rome	7	0.04	100	41_54_N_12_27_E
Spain (Catalunya, Navarra, Extremadura, Aaragón, Cantabria,	4.5	0.0228	4,335	41_22_N_2_11_E
Italy Sardinia pop2	20.6	0.106	1,129	39_14_N_9_3_E
Portugal Azores Terceira Island	1.8	0.0088	130	38_43_N_27_13_W
Greece	31	0.198	48	37_58_N_23_43_E
Greece pop 8	31.3	0.1747	83	37_58_N_23_43_E
Greece pop2	32.6	0.179	120	37_58_N_23_43_E
Greece pop3	30.1		246	37_58_N_23_43_E
Spain Malaga	3.8		160	36_43_N_4_25_W
Spain Malaga Romani	10		80	36_43_N_4_25_W

Box 1 **HLA- class II molecules.**

**Figure 4:**
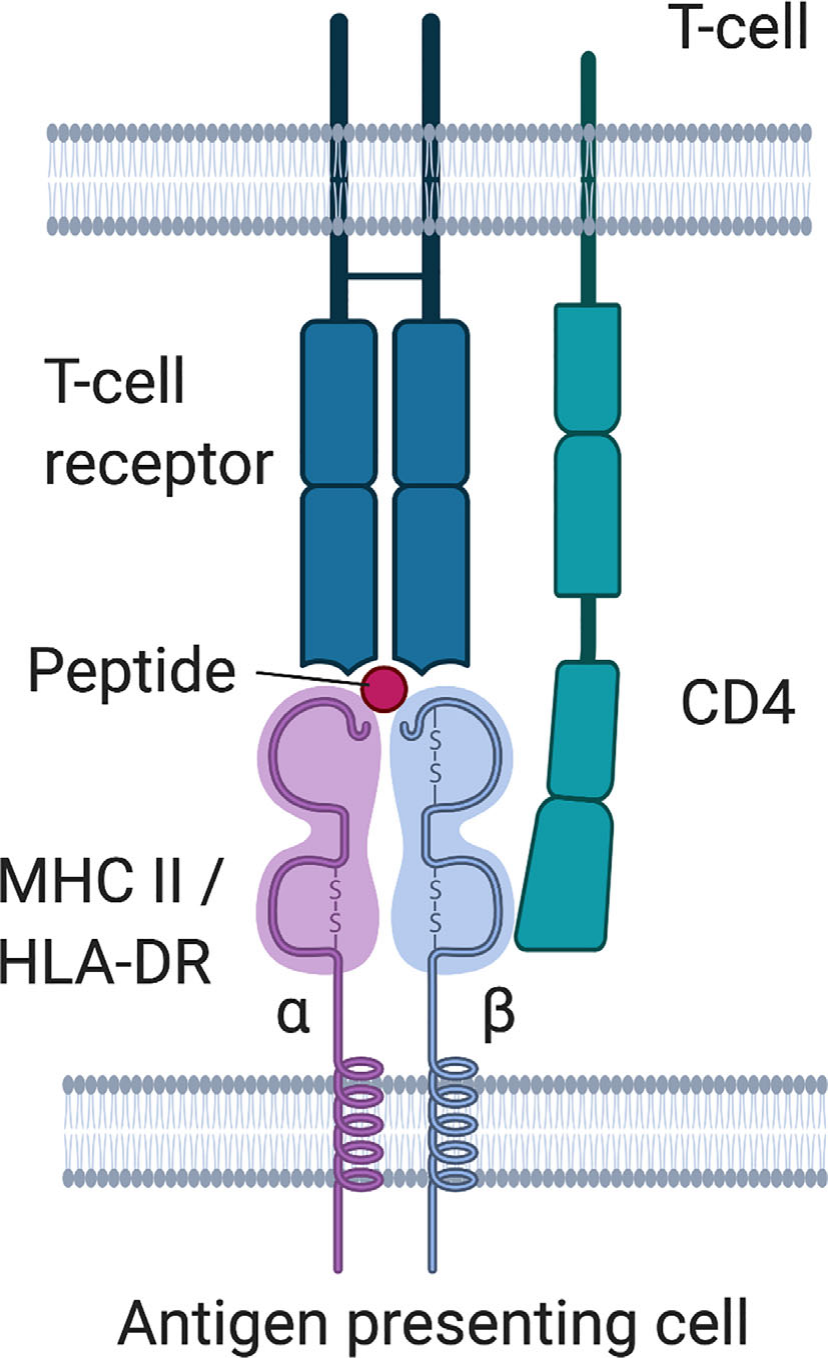
HLA class II molecule structure and interaction with T-cell receptor.

HLA class II molecules are located at the cell surface of professional antigen-presenting cells and epithelial cells in the thymus, and present peptides from extracellular proteins to CD4+ T helper cells or developing T- cells, which then in turn may activate B-cells and stimulate the production of (auto)antibodies. There are three main HLA II molecules, HLA-DR, HLA-DQ and HLA-DP. HLA-DR consists of an α- and a β -chain. The chains consist of two domains, the α1 and α2 as well as the β1 and β2 domain, with the α1 and *β*1 domain forming a groove that presents the peptide. The α chain is encoded by a single gene (DRA1), but the *β* -chain can be coded by four different genes (DRB1, 3, 4, or 5). HLA molecules are highly variable, and HLA-DRB1 is one of the most polymorphic genes with at least 1883 different alleles reported in 2015 (88). Each individual inherits two alleles of DRB1, and additionally variants from DRB3, 4, 5, depending on the parental haplotypes (i.e. whether these genes are present on the parental haplotypes or not). DRB1 is present on all HLA haplotypes, but DRB3, 4, 5 genes are not. HLA-DRB1 is a gene that codes for the *β1* -chain of the DR isotype of the HLA class II molecule (**Figure 4**), and the first field of digits (marked by the semicolon) after the gene name indicate the allele group (alleles with similar sequence and properties), while the second field of digits indicates specific alleles encoding distinct molecule variants. Due to the high variability in the sequence, especially in the peptide binding groove, HLA molecules derived from different alleles generally display distinct peptide repertoires. Therefore, two individuals with different HLA alleles would display different peptides, even from the same protein. HLA-DR also plays a role in central tolerance mechanisms as it has been linked to presentation of autoantigens to developing T-cells in the thymus and is associated with a range of autoimmune diseases, e.g. rheumatoid arthritis, diabetes mellitus type I, multiple sclerosis or systemic lupus erythematosus, suggesting an aberrant presentation of self-peptide to autoreactive T cells in the thymus (95). This may lead to inefficient negative selection of autoreactive T-cells or aberrant education of regulatory T-cells, therefore disrupting central and peripheral tolerance mechanisms and predisposing to autoimmunity. HLA-DRB1 is also associated with HLA-DQB1 due to a strong linkage disequilibrium. An excellent review on HLA-DRB1 and autoimmunity can be found here (88).

## Immunogenic Peptides and how to Present Them: Are There Distinct Shared Sequences in Antigen Peptides and HLA Class II Peptide Binding Grooves?

Next we wanted to investigate whether there is any evidence that may support a functional role of HLA class II molecules for disease susceptibility. One possibility is that distinct HLA class II alleles may play a role in inducing a distinct IgG4 specific T-cell response by the presentation of specific immunogenic peptides (that perhaps are best presented by peptide-binding grooves that have a distinct sequence). This would mean that the HLA haplotype may play a role in the susceptibility to develop IgG4-AIDs *via* specific sequences in the peptide binding groove and/or specific sequences in the presented peptide or in the T-cell receptor (TCR). There is some evidence for this hypothesis.

### HLA Class II Alleles in IgG4-AID

We investigated if there is evidence for any interesting structural characteristics of the peptide-binding groove of IgG4-AID associated HLA class II molecules. Two risk alleles for membranous nephropathy with PLA2R antibodies were identified in a Han Chinese population in DRB1*15:01 and DRB1*03:01 (43), either of which were found in 73.6% of the patient cohort (compared to 33% in the healthy control population), and that contained specific amino acid variants in the peptide-binding groove of the HLA class II molecules which is responsible for antigen presentation to the T-cell receptor (43, 44). Specifically important were arginine at position 13 (arginine_13_) in HLA-DRB1*15:01 and alanine at position 71 (alanine_71_) in DRB1*03:01 for interactions with T cell epitopes of PLA2R. The role of arginine13 in HLA-DRB1*15:01 is also mentioned as relevant in another IgG4-AID. IgG4 autoantibodies against the α3(IV)NC1 in Goodpasture disease also have a strong association with HLA-DRB1*15:01, and that arginine_13_ and proline_11_ of the DRB1*15:01 encoded DR*β*1 chain were associated with disease susceptibility. Furthermore isoleucine_137_, tryptophan_140_, glycine_142_, phenylalanine_143_ and phenylalanine_145_ were presented in peptide-binding pockets 1, 4, 6, 7, and 9, and specifically arginine_13_ in pocket 4 interacts with tryptophan_140_ of the peptide and forms a hydrogen bond (45). Different alleles, HLA-DRB1*04:04,14:02 and 14:06 all share a sequence in the hypervariable region in residues 67 to 74 (LLEQRRAA) and these alleles were reported to be linked to fogo selvagem, an endemic form of pemphigus vulgaris (24). HLA-DRB1*04 is also associated with other IgG4-AID (**Table 1**). In pemphigus, the carrier frequency of the HLA-DRB1*04 allele is very high, for example it is found in 60% of patients from Brazil and in 71% of Dutch patients, with similar and lower allele frequencies in the healthy population in both countries [Brazil: 39%, Netherlands: 38%, (96)]. Analysis of polymorphic residues that form the binding pockets of the DR*β*1 chain of HLA-DRB1*04 and *14 alleles in pemphigus vulgaris show that they contain similar sequences, as well as in HLA-DRB1*04, *14 and *01 in pemphigus foliaceus. Of note are structural similarities between DRB1*04:04 and 01:02 that lead to similar binding pockets and may bind similar peptides [**Table 3**, (25)]. These may present the same peptide from Dsg1, while in pemphigus vulgaris, two distinct types of peptide may be presented by DRB1*04:02 or DRB1*14/04:06. Furthermore, DRB1*14/04:06 may be able to present both Dsg1 and Dsg3 peptides, which may be the reason for antibodies against both antigens in pemphigus vulgaris with muco-cutaneous clinical phenotype (25). Overall it is likely that HLA-DR is important in antigen presentation in pemphigus. HLA- DRB1*04:02 is relevant for the selection of specific self-peptides in pemphigus vulgaris (97), autoreactive T-cells proliferated in response to antigen presented by HLA-DR in Fogo selvagem and this response could be blocked by anti–HLA-DR antibodies, but not by anti–HLA-DQ or anti–HLA-DP antibodies (98).

**Table 3:** Similarities between the two IgG4-AID-associated HLA variants DRB1*0404 and DRB1*0102 in the protein sequence of the two HLA variants (a) and in the amino acid motifs of the peptides bound by these two HLA variants (b).

a) Residues characterizing the binding pockets of DRB1*0404 and DRB1*0102					
DRB1*	P1	P4	P6	P7	P9
0404	V	H Q R A Y	V	**D** Y Y L R	**E** Y **D** Y
0102	V	F Q R A Y	L	**E** C Y L R	W S **D** Y
b) Amino acid motifs of peptides bound to pockets of DRB1*0404 and DRB1*0102					
DRB1*	P1	P4	P6	P7	P9
0404	VLMIF	ALIV/WDEFY/no KR	STNQR	ADHIL/MNPST	ALV/QGSTK
0102	VLMIF	ALIV/MNQ/no HKR	AGSTP		ALV/INFY

Table adapted from (25). Negative charged amino acid (D, E) are in bold, positively charged amino acids (R) are underlined. P1-P9: binding pockets.

### The Role of Peptides Bound to the HLA Class II

If indeed individual HLA class II associations predispose to disease development, could this be mediated by certain immunogenic peptides of the target antigens? Therefore we were interested in the sequences of the IgG4 antigen peptides that are presented by HLA class II molecules and recognized by the T-cell receptor. Several studies investigated T-cell peptides and their (potential) interaction with the binding pockets of the HLA class II, other studies provide at least a list of predicted peptides (**Table 4**). In Goodpasture disease, two overlapping peptides of α3(IV)NC2 that may bind HLA-DRB1*15:01 were investigated: DIPPCPHGWISLWKG and HGWISLWKGFSFIMF, with glycine142 identified as relevant for binding present in both peptides (45, 99). Several Dsg3 peptides binding to HLA-DRB1*04:02 (one of the alleles associated with pemphigus vulgaris) were identified (101–103) and several were validated by *in vitro* tests with patient derived peripheral blood mononuclear cells [PBMCs, (100)]. T-cell responses were elicited by several peptides (**Table 4**), with one peptide, Dsg3190-204 inducing reactivity in all samples and also led to secretion of high levels of IL- 4 and IL-10 (100), which are cytokines for IgG4 class switch.

**Table 4:** An (incomplete) list of predicted and validated peptides of IgG4 autoantigens presented in the MHC II in the context of IgG4-AID identified from published literature.

HLA allele	Peptide	Comment	Antigen	Reference
DRB1*15:01	HGWISLWKGFSFIMF	Tryptophan_140_ forms hydrogen bond with arginine13 in pocket 4, glycine_142_ relevant for binding	α3(IV)NC2	(45)
DRB1*15:01	DIPPCPHGWISLWKG	Cysteine_132_, tryptophan_136_, lysine _141_, and glycine _142_ are relevant for binding	α3(IV)NC2	(99)
DRB1*04:02	LNSKIAFKIVS QEPA	Dsg3_190-204_,Elicited immune response in PBMC of all patients in study, IL-4 and IL-10 secretion	Dsg3	(100)
DRB1*04:02	TPMFLLSRNTGEVRT	Elicited immune response in PBMCs of a subset of patients	Dsg3	(100)
DRB1*04:02	CECNIKVKDVNDNFP		Dsg3	(100)
DRB1*04:02	QSGTMRTRHSTGGTN		Dsg3	(100)
DRB1*04:02	VKILDI NDNPPVFSQQ IFMGEIEENS ASNSLVMILN ATDADEPNHL NS	Induced proliferation of T cells from PV patients	Dsg3	(101)
DRB1*04:02	A DKDGEGLSTQ CECNIKVKDV NDNFPMFRDS QYSARIEENI LSSELLRFQV TDLDEEYTDNWLA		Dsg3	(101)
DRB1*04:02	DSQNNRCEMPR SLTLEVCQCD NRGICGTSYPTTSPGTRYGR PHSG		Dsg3	(101)
DRB1*04:02	IFMGEIEENSASNSLVM	Predicted peptide	Dsg3	(102)
DRB1*04:02	GIAFRPASKTFTVQKGI	Predicted peptide	Dsg3	(103)
DRB1*11:01	GDMLLLWGRLTWRKM	Immunodominant T-cell epitope	ADAMTS13	(104)
DRB1*11/DRB1*15	FINVAPHAR	Core motif presented by DRB1*11 and DRB1*15, recognized by patient CD4+ T cells	ADAMTS13	(105, 106)
HLA-DRB1*11/DRB1*03	ASYILIRD	core motif recognized by CD4+ T-cells	ADAMTS13	(105, 107)
HLA-DQ/DR	IHALATNMG	Predicted peptides presented by HLA-DR	ADAMTS13	(105)
HLA-DR	LIRDTHSLR		ADAMTS13	(105)
HLA-DR	LKTLP-PARC		ADAMTS13	(105)
HLA-DQ/DR	RGPGQADCAVAIGRPLG	Predicted peptide presented by both HLA-DR and -DQ	ADAMTS13	(105)
HLA-DR	SRRQLLSLLSAGRAR	Predicted peptide presented by multiple HLA-DR alleles	ADAMTS13	(105)
HLA-DR	FSEGFLKA-QASLRGQYW		ADAMTS13	(105)
DRB1*11:01	YRFRNKKMK	Predicted core motifs for DRB1*11:01	CASPR2	(39)
	YITLELKKAKLV			
	YFCKMSRLLNT			
	FKTLTPWGV			
	FLGCIRSLRMNGV			
	FLKLDHYPS			
	FNQIAPLKAALR			
	LIRYMFRHKG			
DRB1*07:01	FLFTPSLQLLL	Predicted core motifs for DRB1*07:01	LGI1	(39)
	FRGLKSLIHLSL			
	FVVADSSKA			
	FYSHQSLHA			
	YQWNKATQL			
	FTHVSINKR			
	FLFASSFKGN			

In studies on thrombotic thrombocytopenic purpura (TTP), several T-cell peptides were identified in ADAMTS13 (**Table 4**), with the ADAMTS13 1239-1253 peptide (GDMLLLWGRLTWRKM) as the single immunodominant CD4+ T-cell epitope that was bound both by HLA-DRB1*15:01 and DRB1*11:01 in ELISA and also by T-cells from a strain of mice expressing human HLA-DRB1*01:01 (104). A recent study looking into peptides presented on HLA-DR and HLA-DQ (105) identified 12 peptides presented by DR and 8 by DQ. The study identified an overlap and also differences in the repertoire of ADAMTS13 peptides that are presented on HLA-DQ and HLA-DR. Most DR peptides derived from the CUB-domain of ADAMTS13. DQ-presented peptides mostly derived from the cysteine-rich, TSP2, and TSP2-linker 1 of ADAMTS13, which demonstrates that haplotypes have distinct peptide repertoires. Nevertheless there are also peptides which could be presented by both DR and DQ (**Table 4**). Furthermore, polymorphisms in the antigen may play a role in disease susceptibility. In pemphigus foliaceus a coding synonymous T/C single nucleotide polymorphism (SNP) at position 809 in the DSG1 gene was found in French patients (108). This mutation is silent, but the SNP was hypothesized to have a role in alternative splicing (109). In a different study, alternative splicing of DSG1 led to a 101-bp insertion at the 3′ end of *DSG1*-intron 6 and introducing a stop codon in the nucleotide sequence, thus coding for a truncated isoform of Dsg1 containing a peptide that was presented by DRβ1*01:02 and activated autoreactive T-cells (110).

### Is There Evidence for Selective T-Cell Receptor VDJ Usage?

Peptides presented in the HLA class II molecules are recognized by the T-cell receptor (TCR). Therefore we also wanted to study whether the structure of the TCR may play a role for the recognition of IgG4 antigens, and therefore investigated the literature for evidence of specific TCR VDJ usage (which directly would influence structure of the TCR) in MuSK MG and pemphigus foliaceus. There was no evidence for a preference for a specific Vβ gene in pemphigus foliaceus (111), but in pemphigus vulgaris, different Vβ genes were used, with cells reacting to peptide Dsg3(AA145-192/VKILDI NDNPPVFSQQ IFMGEIEENS ASNSLVMILN ATDADEPNHL NS), (AA240-303, A DKDGEGLSTQ CECNIKVKDV NDNFPMFRDS QYSARIEENI LSSELLRFQV TDLDEEYTDN WLA) or (AA570-614, D SQNNRCEMPR SLTLEVCQCD NRGICGTSYP TTSPGTRYGR PHSG) being linked to Vβ13, 7 or 17 genes, respectively, and Dsg3(AA145-192) and (AA240-303) were also linked to Vα22 or 10, respectively (112).In a study with 13 MuSK MG patients, MuSK responsive T-cells from HLA-DQB1*05+ MuSK-MG patients had a restricted set of TCR VJ rearrangements. Two common motifs in TRBV29 were identified as GXGQET and TEHQET, these were shared in 4 patients (113). There is not enough data available to make any assumptions yet, and overall there is a need for more studies investigating the TCR-peptide-HLA class II interaction in the different IgG4-AID.

## Hypothetical Etiology and Mechanisms in IgG4-Autoantibody Associated Diseases: A Unifying Theory?

The HLA class II gene region is linked to immunopathogenesis of autoimmune diseases (88, 114, 115) and we are specifically interested whether a subset of HLA alleles may be specifically linked with IgG4-autoimmunity. The presentation of autoantigens to developing T-cells relies strongly on the highly polymorphic β chain of HLA-DR (**Box 1**). Perhaps the structure of the peptides presented by HLA-DR *β*1 variants contribute to the generation of IgG4 autoantibodies later on by aberrantly priming T-cells, by the type of peptide that is presented, by the conformation of the peptide in the peptide-binding groove or by altered interaction with the T-cell receptor or CD4. In line with this theory is the observation of one study that risk alleles HLA-DRB1*15:01 and HLA-DRB3*02:02 for PLA2R-IgG4 associated MN (DRB1*15:01 also being associated with further IgG4 AID such as Goodpasture disease or TTP, **Table 1**) contain specific amino acid variants in the peptide-binding groove of the DR *β* chain responsible for antigen presentation to the TCR (43).

**Figure 5:**
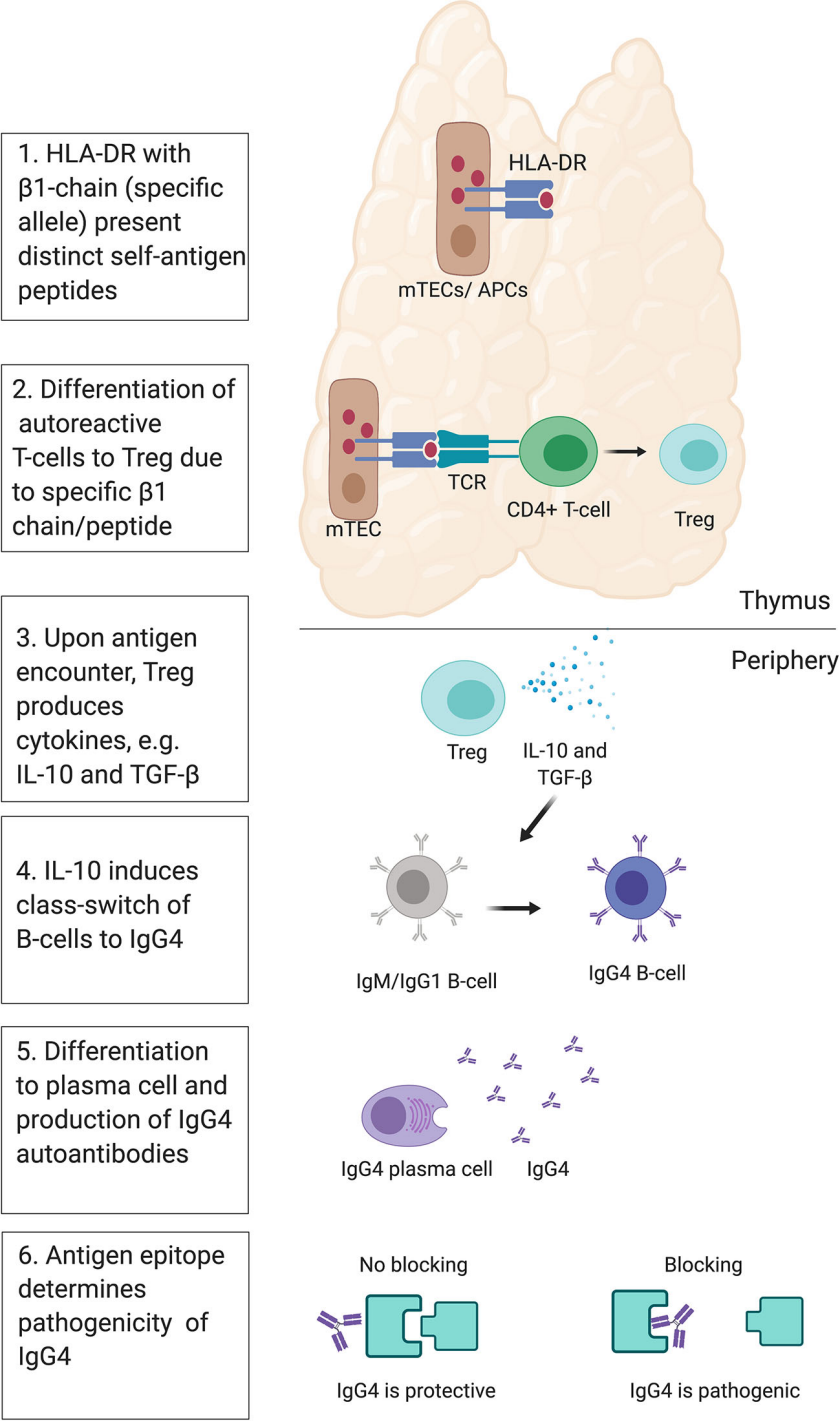
Theory of IgG4 induction. Presentation of distinct self- peptides *via* HLA class II in medullary thymic epithelial cells (mTECs) or antigen-presenting cells (APCs) to developing autoreactive CD4+ T-cells *via* the T-cell receptor (TCR) may influence their fate toward differentiation into regulatory T-cells (Tregs). This may be influenced by polymorphisms in the beta-chain of the HLA-DR molecule, and/or the type of peptide presented to the TCR. In the periphery, Tregs may respond to their autoantigen by producing anti-inflammatory cytokines IL-10 and TGF-beta. IL-10 may then induce a class switch of autoreactive B-cells toward IgG4. The B-cell may then differentiate into plasma cells, producing autoreactive IgG4. IgG4 then bind their self-antigen, and depending on the location of the epitope, may cause a blocking effect and induce pathogenicity or not.

The HLA is responsible for the fate of T-cells, e.g. conventional pro-inflammatory T cells (Tconvs) or regulatory T cells (Tregs). A recent study with humanized mice investigated the T-cell response to self-antigen presented by HLA-DR molecules with different β chain alleles, HLA-DR1 or HLA-DR15 in mice (116). The two HLA class II molecules presented distinct peptide repertoires and the presentation *via* HLA-DR15 was associated with the generation of Tconv, while presentation *via* HLA-DR1 instead led to the development of Tregs that expressed tolerogenic cytokines such as IL-10 and TGF- β (116). Similar conclusions were drawn from a different study where mouse MHC II alleles (H2-Aj and H2-Ab) influenced the T-cell repertoire, including Treg and Tconv (117). This links the polymorphism in the HLA-DR molecules to the fate of T-cells and differentiation to Tregs and production of IgG4 *via* IL-10 (118–121). Moreover, blocking of DR15 prevented onset and attenuated severity of disease in animals in a follow-up study (122), suggesting that the HLA class II antigen presentation pathway has potential as a novel therapeutic target. Under normal circumstances, the generation of Tregs and a class switch of autoantibodies to IgG4 would protect from autoimmunity. IL-10 mediated class switch to protective IgG4 has been demonstrated in the context of allergen immunotherapy (123). In non-IgG4 autoimmune diseases, there may be a failure in this central tolerance mechanisms, leading to the survival of autoreactive CD4+ T-cells and in the production of non-IgG4 autoantibodies. This suggests that in IgG4-AID, a normally protective tolerance mechanism is at work, and the pathogenic effects of blocking IgG4 may just be an unlucky side effect (**Figure 5**).

## Potential Factors Influencing IgG4 Antibody Production

IgG4 subclass autoantibodies are the main cause of IgG4-AID and directly pathogenic, Therefore we were interested whether there is evidence for a mechanism linking specific HLA class II alleles to Tregs to IL-10 and finally to IgG4 autoantibodies. There are contradictory observations. Supporting evidence: 1) MuSK MG patients with the HLA-DRB1*14 type had elevated antibody titers and higher levels of IL-17A and IL-10 than MuSK MG patients with different haplotype (124). The higher IgG4 titers of MuSK autoantibodies in patients carrying HLA-DRB1*14 than those in the other patients suggest a role for HLA in the production of the antibodies. The differences in IL-10 and IL-17A support the role of DRB1 in the etiopathogenesis of this autoimmune response (124).

2) In addition, active immunization of mice with MuSK antigen leads to production of MuSK antibodies predominantly of the IgG1 subclass (which is the mouse equivalent to human IgG4), IL-4 (which induces IgG1- production in mice) and IL-10 (125). 3) Also IL-4 and IL-10 levels were increased in an adoptive transfer model of pemphigus vulgaris (126), and 4) IL-10 levels are elevated in serum of pemphigus patients (127, 128), serum of MuSK-MG patients (129) and serum of patients with thrombotic thrombocytopenic purpura (130), but in the latter study the IgG subclass levels were not entirely clear, with some patients also demonstrating high levels of complement fixing antibodies and complement C3a and C5a, suggesting that IgG4 was not the predominant subclass. More evidence for a role of IL-10 comes from an association between genetic variants/haplotypes of IL-10 and the occurrence of pemphigus vulgaris in patients from Argentina and Slovakia (131, 132).

What is not in line with the hypothesis that the DR haplotype directly leads to increased IgG4 antibody levels is that one study of TTP showed increased ADAMTS13 antibody levels in patients with the protective DR7-DQ2 and DR13-DQ6 haplotypes, though the relevance is not clear (133). Furthermore, there is evidence against the hypothesis that increased Treg and IL-10 levels (measured in the blood) may lead to IgG4 class switch and production of pathogenic IgG4 autoantibodies, as 1) total Treg numbers were not increased in MuSK MG (134), even decreased in pemphigus vulgaris [while Th17 T-cells were increased (135)] and 2) IL-10+ antigen-specific Tregs are only found in 17% of patients with pemphigus vulgaris, but in 80% of healthy controls, which suggests (at least in pemphigus) a protective role of Tregs and IL-10 (136). 2) B-cells from MuSK patients did not produce increased levels of IL-10 in culture (129, 137). 3) Further studies suggest a protective role of IL-10 in pemphigus, as IL-10^−/−^ mice were more susceptible to disease and IL-10 protected mice from blister formation (138), and decreased IL-10 production was associated with pemphigus vulgaris in a subgroup of patients (131). 4) Also numbers of IL-10 producing Bregs (B10 cells) were reduced in MuSK MG patients (139), as well as pemphigus patients in one study (140), but increased in pemphigus patients in another study (141). 5) Interestingly, increased levels of IL-10 producing Bregs was associated with favorable response after B-cell depletion therapy with Rituximab in pemphigus (142), which may indicate a protective role for IL-10 and Bregs, but this could also have been an unrelated side-effect of Rituximab-induced increase in cytokines that stimulate IL-10 production (143, 144).

Taken together, there may not be a simple correlation between Tregs, Bregs, IL-10 and IgG4 autoantibodies, since IL-10 does have multiple function in immune regulation, and may have both anti- and pro-inflammatory effects (145), also in the context of IgG4-autoimmunity (146). Perhaps not increased systemic levels of IL-10, but local expression of IL-10 is important, as seen in studies on rheumatoid arthritis, where local administration of IL-10 to sites of inflammation was protective (147), but systemic application was not (148). More studies are required, and ideally also a systematic review and meta-analysis of cytokine levels in IgG4 autoimmune diseases and/or disease models.

## Conclusion

In recent years, we could observe a growing number of rare, severe autoimmune diseases mediated by pathogenic autoantibodies of the IgG4 subclass. While these diseases were previously considered to be unrelated, as they may target different organs, there is (at least circumstantial) evidence that ties them together and suggests that similar immune mechanisms may be at work. These potential mechanisms need to be addressed in large comparative studies, and, once identified, may form the basis for new treatment approaches. These may provide a rationale for repurposing already existing drugs and treatment strategies used in well-known diseases such as MuSK MG or pemphigus for treatment of the very rare and the less-well studied forms of IgG4 autoimmune diseases. Newly identified shared immune mechanisms may also provide new druggable targets and ideas for novel treatments.

## Author Contributions

IK conceived the idea, wrote the first draft of the manuscript, and provided the figures. VY, JT, KL, ST, ET, and TL contributed text. IK, FL, TL, and ET revised the final manuscript. All authors contributed to the article and approved the submitted version.

## Funding

IK was funded by a Hertha Firnberg Fellowship by the Austrian Science Fund, Austria, project number: T996-B30. TL was supported by the German Research Foundation (DFG, grant LE 2593/3-1). FL is supported by German Ministry of Education and Research (01GM1908A) and E-Rare Joint Transnational research support (ERA-Net, LE3064/2-1).

## Conflict of Interest

JT and ST have shares in the research and diagnostic laboratory Tzartos NeuroDiagnostics, Athens. FL discloses having received speaker honoraria from Grifols, Teva, Biogen, Bayer, Roche, Novartis, Fresenius, travel funding from Merck, Grifols, and Bayer and serving on advisory boards for Roche, Biogen, and Alexion.

The remaining authors declare that the research was conducted in the absence of any commercial or financial relationships that could be construed as a potential conflict of interest.
