# Supplementary material for: Common Denominators in the Immunobiology of IgG4 Autoimmune Diseases: What Do Glomerulonephritis, Pemphigus Vulgaris, Myasthenia Gravis, Thrombotic Thrombocytopenic Purpura and Autoimmune Encephalitis Have in Common?
Source: Front Immunol. 2021 Jan 29;11:605214. doi: 10.3389/fimmu.2020.605214 (PMC7878376; doi:10.3389/fimmu.2020.605214)
Supplement: Supplementary file 1 [file Table_1.docx]

Supplementary table 1: Results from human genome-wide association studies on IgG4 autoimmune diseases. Summary data were downloaded from the NHGRI-EBI GWAS Catalog (1)on 12/11/2020.

| IgG4 autoimmune disease | IgG4 classification | Mapped gene | P-value | Study accession |
| --- | --- | --- | --- | --- |
| Pemphigus vulgaris | Class I | RNU2-40P, AC104137.1 | 1 x 10-6 | GCST006521 |
| Pemphigus vulgaris | Class I | MIR3660, AC113167.1 | 2 x 10-6 | GCST006521 |
| Pemphigus vulgaris | Class I | THSD7B | 8 x 10-6 | GCST006521 |
| Pemphigus vulgaris | Class I | PPP1R10 | 5 x 10-6 | GCST006521 |
| Pemphigus vulgaris | Class I | MTCO3P1, HLA-DQB1 | 4 x 10-19 | GCST008068 |
| Pemphigus vulgaris | Class I | HLA-DRB1, HLA-DQA1 | 1 x 10-45 | GCST008068 |
| Pemphigus vulgaris | Class I | HLA-DQB1, HLA-DQA1 | 3 x 10-35 | GCST006584 |
| Pemphigus vulgaris | Class I | RNU6-592P, GLULP3 | 3 x 10-8 | GCST006584 |
| Pemphigus vulgaris | Class I | AL662789.1, MTCO3P1 | 3 x 10-12 | GCST006584 |
| Pemphigus vulgaris | Class I | HLA-DRA | 3 x 10-6 | GCST006584 |
| Pemphigus foliaceus | Class I | AC073912.1, RAN | 2 x 10-9 | GCST006520 |
| Pemphigus foliaceus | Class I | HLA-V, AL645939.4 | 7 x 10-9 | GCST006520 |
| Membranous glomerulonephritis | Class II | PLA2R1 | 4 x 10-10 | GCST003402 |
| Membranous glomerulonephritis | Class II | HLA-DQA1 | 2 x 10-39 | GCST003402 |
| Membranous glomerulonephritis | Class II | HLA-DQA1 | 8 x 10-93 | GCST000984 |
| Membranous glomerulonephritis | Class II | HLA-B | 6 x 10-72 | GCST000984 |
| Membranous glomerulonephritis | Class II | TSBP1-AS1, TSBP1 | 5 x 10-71 | GCST000984 |
| Membranous glomerulonephritis | Class II | STK19 | 8 x 10-66 | GCST000984 |
| Membranous glomerulonephritis | Class II | TCF19 | 3 x 10-58 | GCST000984 |
| Membranous glomerulonephritis | Class II | C6orf15, RNU6-1133P | 9 x 10-58 | GCST000984 |
| Membranous glomerulonephritis | Class II | PRRC2A | 7 x 10-57 | GCST000984 |
| Membranous glomerulonephritis | Class II | PRRC2A | 1 x 10-56 | GCST000984 |
| Membranous glomerulonephritis | Class II | GPANK1 | 2 x 10-56 | GCST000984 |
| Membranous glomerulonephritis | Class II | BTNL2, TSBP1-AS1 | 1 x 10-48 | GCST000984 |
| Membranous glomerulonephritis | Class II | PSORS1C1, C6orf15 | 6 x 10-48 | GCST000984 |
| Membranous glomerulonephritis | Class II | EHMT2, EHMT2-AS1 | 3 x 10-46 | GCST000984 |
| Membranous glomerulonephritis | Class II | PSORS1C1 | 1 x 10-42 | GCST000984 |
| Membranous glomerulonephritis | Class II | EGFL8, PPT2-EGFL8 | 1 x 10-41 | GCST000984 |
| Membranous glomerulonephritis | Class II | RNF5 | 2 x 10-40 | GCST000984 |
| Membranous glomerulonephritis | Class II | TSBP1, TSBP1-AS1 | 2 x 10-40 | GCST000984 |
| Membranous glomerulonephritis | Class II | SFTA2, MUCL3, HCG21 | 3 x 10-40 | GCST000984 |
| Membranous glomerulonephritis | Class II | PRRT1, FKBPL | 9 x 10-40 | GCST000984 |
| Membranous glomerulonephritis | Class II | AL645941.2 | 4 x 10-39 | GCST000984 |
| Membranous glomerulonephritis | Class II | POU5F1 | 4 x 10-37 | GCST000984 |
| Membranous glomerulonephritis | Class II | PLA2R1 | 9 x 10-29 | GCST000984 |
| Membranous glomerulonephritis | Class II | PLA2R1 | 5 x 10-103 | GCST010004 |
| Membranous glomerulonephritis | Class II | NFKB1 | 3 x 10-12 | GCST010004 |
| Membranous glomerulonephritis | Class II | IRF4, DUSP22 | 1 x 10-14 | GCST010004 |
| Membranous glomerulonephritis | Class II | HLA-DQA1, HLA-DRB1 | 3 x 10-154 | GCST010004 |
| Membranous glomerulonephritis | Class II | PLA2R1 | 5 x 10-48 | GCST010005 |
| Membranous glomerulonephritis | Class II | NFKB1 | 8 x 10-7 | GCST010005 |
| Membranous glomerulonephritis | Class II | HLA-DQA1, HLA-DRB1 | 2 x 10-60 | GCST010005 |
| Membranous glomerulonephritis | Class II | PLA2R1 | 4 x 10-61 | GCST010006 |
| Membranous glomerulonephritis | Class II | NFKB1 | 2 x 10-61 | GCST010006 |
| Membranous glomerulonephritis | Class II | IRF4, DUSP22 | 9 x 10-14 | GCST010006 |
| Membranous glomerulonephritis | Class II | HLA-DQA1, HLA-DRB1 | 4 x 10-102 | GCST010006 |
| Eosinophilic granulomatosis with polyangiitis | Class III | AC114798.1 | 2 x 10-6 | GCST002160 |
| Eosinophilic granulomatosis with polyangiitis | Class III | AC012441.1, AC005999.2 | 4 x 10-7 | GCST002160 |
| Eosinophilic granulomatosis with polyangiitis | Class III | AP000777.1, AP000777.2 | 2 x 10-7 | GCST002160 |
| Eosinophilic granulomatosis with polyangiitis | Class III | RNU6-1264P, BTF3P14 | 2 x 10-7 | GCST002160 |
| Eosinophilic granulomatosis with polyangiitis | Class III | SEMA6A, COMMD10 | 2 x 10-8 | GCST002159 |
| Eosinophilic granulomatosis with polyangiitis | Class III | HLA-DPB1 | 2 x 10-50 | GCST002160 |
| Eosinophilic granulomatosis with polyangiitis | Class III | HLA-DQB1, MTCO3P1 | 1 x 10-41 | GCST009248 |
| Eosinophilic granulomatosis with polyangiitis | Class III | RPL7P38, RNU7-106P | 1 x 10-8 | GCST009248 |
| Eosinophilic granulomatosis with polyangiitis | Class III | GPA33 | 7 x 10-10 | GCST009249 |
| Eosinophilic granulomatosis with polyangiitis | Class III | HLA-DQB1, MTCO3P1 | 3 x 10-9 | GCST009249 |
| Eosinophilic granulomatosis with polyangiitis | Class III | ACOXL-AS1, ACOXL, MIR4435-2HG | 9 x 10-11 | GCST009250 |
| Eosinophilic granulomatosis with polyangiitis | Class III | TSLP, AC010395.1 | 5 x 10-11 | GCST009250 |
| Eosinophilic granulomatosis with polyangiitis | Class III | HLA-DQB1, MTCO3P1 | 1 x 10-20 | GCST009250 |
| Eosinophilic granulomatosis with polyangiitis | Class III | AC044784.1, LINC00709 | 3 x 10-8 | GCST009250 |
| Eosinophilic granulomatosis with polyangiitis | Class III | GNE, CLTA | 1 x 10-6 | GCST009999 |
| Eosinophilic granulomatosis with polyangiitis | Class III | PMM2 | 3 x 10-6 | GCST009999 |
| Eosinophilic granulomatosis with polyangiitis | Class III | AC104465.1, AC092810.4 | 9 x 10-6 | GCST009998 |
| Eosinophilic granulomatosis with polyangiitis | Class III | AC019211.1, AC093083.1 | 5 x 10-6 | GCST009998 |
| Eosinophilic granulomatosis with polyangiitis | Class III | GAPDHP50, ICE2P2 | 5 x 10-6 | GCST009998 |
| Eosinophilic granulomatosis with polyangiitis | Class III | CPEB2, C1QTNF7-AS1 | 4 x 10-6 | GCST009998 |
| Eosinophilic granulomatosis with polyangiitis | Class III | MCUB | 6 x 10-6 | GCST009998 |
| Eosinophilic granulomatosis with polyangiitis | Class III | MRPL36 | 6 x 10-6 | GCST009998 |
| Eosinophilic granulomatosis with polyangiitis | Class III | COL19A1 | 2 x 10-6 | GCST009998 |
| Eosinophilic granulomatosis with polyangiitis | Class III | FGF19 | 5 x 10-6 | GCST009998 |
| Eosinophilic granulomatosis with polyangiitis | Class III | OR10D3, OR8F1P | 4 x 10-6 | GCST009998 |
| Eosinophilic granulomatosis with polyangiitis | Class III | TSPAN9 | 9 x 10-6 | GCST009998 |
| Eosinophilic granulomatosis with polyangiitis | Class III | KRT73-AS1 | 2 x 10-6 | GCST009998 |
| Eosinophilic granulomatosis with polyangiitis | Class III | HIF3A, IGFL1P2 | 7 x 10-6 | GCST009998 |
| Eosinophilic granulomatosis with polyangiitis | Class III | RHOU, AL078624.2 | 4 x 10-7 | GCST010000 |
| Eosinophilic granulomatosis with polyangiitis | Class III | CTNNA2 | 3 x 10-6 | GCST010000 |
| Eosinophilic granulomatosis with polyangiitis | Class III | NBPF21P, RN7SKP227 | 4 x 10-6 | GCST010000 |
| Eosinophilic granulomatosis with polyangiitis | Class III | AC106799.2 | 6 x 10-6 | GCST010000 |
| Eosinophilic granulomatosis with polyangiitis | Class III | AC034244.1, AC034244.3 | 6 x 10-6 | GCST010000 |
| Eosinophilic granulomatosis with polyangiitis | Class III | LCP2 | 2 x 10-6 | GCST010000 |
| Eosinophilic granulomatosis with polyangiitis | Class III | PPM1AP1, TUSC3 | 8 x 10-6 | GCST010000 |
| Eosinophilic granulomatosis with polyangiitis | Class III | AC083967.1, C8orf34 | 4 x 10-7 | GCST010000 |
| Eosinophilic granulomatosis with polyangiitis | Class III | CELF2-DT | 3 x 10-6 | GCST010000 |
| Eosinophilic granulomatosis with polyangiitis | Class III | PWRN1 | 9 x 10-6 | GCST010000 |
| Eosinophilic granulomatosis with polyangiitis | Class III | HS3ST3A1, LINC02093 | 4 x 10-6 | GCST010000 |
| Eosinophilic granulomatosis with polyangiitis | Class III | NFKBIB | 7 x 10-7 | GCST010000 |
| Eosinophilic granulomatosis with polyangiitis | Class III | RN7SL555P, AVP | 4 x 10-6 | GCST010000 |
| Eosinophilic granulomatosis with polyangiitis | Class III | TGM2 | 8 x 10-6 | GCST010000 |

1. Buniello A, MacArthur JAL, Cerezo M, Harris LW, Hayhurst J, Malangone C, et al. The NHGRI-EBI GWAS Catalog of published genome-wide association studies, targeted arrays and summary statistics 2019. Nucleic Acids Res. 2019;47(D1):D1005-D12. doi: 10.1093/nar/gky1120
